# Chronic Salsolinol Administration Prevents the Behavioral and Neurochemical Effects of l-DOPA in Rats

**DOI:** 10.1007/s12640-015-9523-2

**Published:** 2015-02-25

**Authors:** Agnieszka Wąsik, Irena Romańska, Jerzy Michaluk, Lucyna Antkiewicz-Michaluk

**Affiliations:** Department of Neurochemistry, Institute of Pharmacology, Polish Academy of Sciences, 12 Smetna Street, 31-343 Kraków, Poland

**Keywords:** 1-Methyl-6,7-dihydroxy-1,2,3,4-tetrahydroisoquinoline (salsolinol), l-DOPA, Parkinson’s disease, Microdialysis study, Dopamine

## Abstract

1-Methyl-6,7-dihydroxy-1,2,3,4-tetrahydroisoquinoline (salsolinol) is a well-known endogenous compound that has been proposed as a factor involved in the pathogenesis of Parkinson’s disease. In the present study, we investigated the impact of acute and chronic salsolinol (100 mg.kg i.p.) administration on l-DOPA-induced locomotor hyperactivity and neurochemical changes (the dopamine level and its metabolism in rat brain structures). Moreover, using the in vivo microdialysis technique, we measured the effect of acute and chronic salsolinol injection on l-DOPA-induced dopamine release in the rat striatum. The behavioral data demonstrated that both acute and chronic salsolinol administration antagonized l-DOPA-mediated hyperactivity. An ex vivo neurochemical experiment indicated that chronic but not acute salsolinol administration partially inhibited the l-DOPA-induced increases in the concentration of dopamine and all of its metabolites in dopaminergic structures. Additionally, the in vivo dopamine release data obtained from the microdialysis experiments clearly indicated that the differences in the effect of salsolinol on the activities of l-DOPA depended on the mode of salsolinol treatment. Acute injection of salsolinol enhanced the l-DOPA-induced elevation of dopamine release (by ~1200 %; *P* < 0.01), whereas chronic administration of salsolinol completely blocked the l-DOPA-induced elevation of dopamine release in the rat striatum. These data demonstrated that chronic administration of salsolinol significantly impaired the response of dopaminergic neurons to l-DOPA administration. In conclusion, we propose that an elevated salsolinol level in parkinsonian patients may represent a serious risk factor of the clinical efficacy of l-DOPA therapy.

## Introduction

The primary pathological process that characterizes Parkinson’s disease (PD) is the progressive degeneration of dopaminergic neurons in the nigro-striatal pathway. This process leads to a dramatic decrease in the dopamine concentration in the substantia nigra. The classical cardinal signs of PD are rigidity, bradykinesia, resting tremor, and postural instability. Oral administration of the dopamine precursor l-DOPA has become the gold standard for the treatment of PD. Although available medications provide some symptomatic relief to nearly all patients, none of these drugs have been shown to significantly slow or halt disease progression (Lang 2009; Fernandez 2012). Recent investigations of PD etiopathology suggest that the neurodegeneration that occurs in this disease involves several cellular and molecular events, such as oxidative stress, microglia-mediated inflammation, and proapoptotic mechanisms (von Bohlen et al. 2004). 1-Methyl-6,7-dihydroxy-1,2,3,4-tetrahydroisoquinoline (salsolinol) is an endogenous tetrahydroisoquinoline derivative which has been found to induce slowly developing neurodegenerative changes (Lorenc-Koci et al. 2008; Wąsik et al. 2009, 2014). In vivo, salsolinol can be formed in the mammalian brain via different mechanisms, e.g., the selective synthesis of salsolinol from dopamine and acetaldehyde by salsolinol synthase (Maruyama et al. 1997; Naoi et al. 1996). Salsolinol is present in the human brain, particularly in alcoholics. High levels of this catechol have been measured in dopaminergic brain structures such as the substantia nigra and the striatum (Naoi et al. 2002). Additionally, an elevated concentration of salsolinol was found in the cerebrospinal fluid of parkinsonian patients, and its concentration positively correlated to the degree of motor disability and dementia (Antkiewicz-Michaluk et al. 1997). Salsolinol has been proposed as etiological factor of PD (Moser and Kompf 1992; Antkiewicz-Michaluk et al. 2000a, b). Weiner and Collins (1978) demonstrated that salsolinol inhibits tyrosine hydroxylase, a rate-limiting enzyme in dopamine synthesis, isolated from the rat brain. Chronic administration of salsolinol induces the degeneration of striatal dopaminergic neurons (Antkiewicz-Michaluk et al. 2000b). Our previous experiments have shown that salsolinol antagonized the biochemical and behavioral effects of apomorphine but did not potentiate the cataleptogenic activity of haloperidol, as salsolinol displayed affinity to binding sites labeled with [H3] apomorphine but did not displace dopamine receptor antagonists (Antkiewicz-Michaluk et al. 2000a). As demonstrated by in vitro studies, salsolinol reduces cell viability and both BDNF- and p-CREB-mediated signal transduction protein in a dose-dependent manner. Furthermore, salsolinol induces a significant increase in the activity of caspase-3 in dopaminergic SH-SY5Y cells (Brown et al. 2013).

The aim of the present study was to examine the effects of acute and chronic administration of salsolinol on l-DOPA-mediated activities using both behavioral and neurochemical studies in rats. To this end, we examined the effect of salsolinol on l-DOPA-induced locomotor hyperactivity, dopamine release in the striatum, and changes in dopamine metabolism in different brain structures in vivo.

## Materials and Methods

### Animals and Treatment

The experiments were performed on male Wistar rats whose initial body weight was 220–240 g. All animals were provided with free access to standard laboratory food and tap water and were maintained at room temperature (22 °C) under an artificial light/dark cycle (12/12 h, lights on at 7 a.m.).

The rats were administered with salsolinol at a dose of 100 mg/kg intraperitoneally (i.p.*)* once or chronically for 14 consecutive days. In the combined treatment group, l-DOPA (100 mg/kg i.p.) was administered once 15 min after the final salsolinol administration. Control rats were treated with an appropriate solvent. The rats were euthanized via decapitation 2 h after the final drug injection, and different structures of the brain were dissected. These experiments were performed between 9:00 and 16:00.

All procedures were performed in accordance with the National Institutes of Health Guide for the Care and Use of Laboratory Animals and were approved by the Bioethics Commission in compliance with Polish law. All experimental procedures were approved by the Local Bioethics Commission of the Institute of Pharmacology of the Polish Academy of Sciences in Kraków.

### Drugs

Salsolinol and l-DOPA (Sigma-Aldrich, USA) were obtained commercially. These compounds were dissolved in a 0.9 % NaCl solution.

### Behavioral Study

#### Locomotor Activity

Locomotor activity was examined using actometers Opto-Varimex activity monitors (Columbus Inst., USA) linked on-line to a compatible IBM-PC. Each cage (43 × 44 × 25 cm) was surrounded by a 15 × 15 array of photocell beams located 3 cm from the floor surface as reported previously (Filip et al. 2007). The interruptions in these photocell beams were counted as a measure of horizontal locomotor activity, which was defined as the distance traveled (in cm). Horizontal locomotor activity was recorded for 30 min and analyzed using Auto-Track Software (Columbus Instruments, USA). The animals were placed in the actometers, and after 40 min of adaptation, the animals received the specified drugs. The rats received salsolinol at a dose of 100 mg/kg i.p. acutely or chronically for 14 consecutive days. Additionally, l-DOPA (100 mg/kg i.p.) was acutely administered 15 min after salsolinol administration; the control group was treated with saline. Horizontal locomotor activity was assessed for 30 min. Six animals per group were used.

### Biochemical Studies

#### Ex Vivo Experiments

##### Dopamine Metabolism and l-DOPA Metabolism

Two hours after the final salsolinol injection, the rats were euthanized via decapitation, and the substantia nigra and striatum were immediately dissected. The obtained tissue was frozen on dry ice (−70 °C) until further use for biochemical assays. Dopamine, its metabolites 3,4-dihydroxyphenylacetic acid (DOPAC), 3-methoxytyramine (3-MT), and homovanillic acid (HVA), and the l-DOPA metabolite 3-methoxy-DOPA (3-MDOPA) were measured via high-performance liquid chromatography (HPLC) using an electrochemical detection system (Hewlett Packard 1049A). The tissue samples were weighed and homogenized in ice-cold 0.1 M perchloroacetic acid containing 0.05 mM ascorbic acid. After centrifugation (10,000*g*, 5 min), the supernatants were filtered through RC 58 0.2-µm cellulose membranes (Bioanalytical Systems, West Lafayette, IN, USA). The HP 1050 chromatograph (Hewlett-Packard, Golden, CO, USA) was equipped with C18 columns. The mobile phase consisted of 0.05 M citrate–phosphate buffer (pH 3.5), 0.1 mM EDTA, 1 mM sodium octyl sulfonate, and 3.5 % methanol. The flow rate was maintained at 1 ml/min. The potential was 800 mV. Dopamine and its metabolites were quantified via comparison to the peak heights of the corresponding standards measured on the day of analysis. Seven to ten animals per group were used.

### In Vivo Microdialysis Study and Analytical Procedure

The rats were anesthetized using ketamine (75 mg/kg) and xylazine (10 mg/kg) and secured in a stereotaxic frame (Stoelting, USA). Vertical microdialysis guides (Intracerebral Guide Cannula with stylet; BAS Bioanalytical, USA) were implanted in the striatum (STR) at the following stereotaxic coordinates: A/P +1.0 and L/M +2.5 from Bregma and V/D −3.5 mm from the dura (G. Paxinos and CH Watson). On the seventh day after surgery, the microdialysis probes were placed inside the guides, and the striatum was perfused with artificial cerebrospinal fluid (aCSF) consisting of 140 mM NaCl, 2.7 mM KCl, 1.2 mM CaCl_2,_ 1 mM MgCl_2_, 0.3 mM NaH_2_PO_4_, and 1.7 mM Na_2_HPO_4_, pH 7.4, at a flow rate of 1.5 μl/min using a microinfusion pump (Stoelting, IL, USA). Samples were collected from freely moving rats at 20 min intervals after a 3 h wash-out period. Salsolinol was injected i.p. (acutely or chronically for 14 consecutive days) at a 50 mg/kg dose, and the samples were collected for 180 min. In the combined treatment groups, l-DOPA (100 mg/kg i.p.) was administered once 40 min after final salsolinol administration. All probes were immediately frozen on dry ice (−70 °C) until use for biochemical assays.

Dopamine (DA) was measured in dialysates (20 μl) via HPLC using an electrochemical detection system. The HP1050 chromatograph (Hewlett-Packard, Golden, CO, USA) was equipped with C18 columns. The mobile phase consisted of 0.05 M citrate–phosphate buffer, pH 3.5, 0.1 mM EDTA, 1 mM sodium octyl sulfonate, and 3.5 % methanol. The flow rate was 1 ml/min.

The chromatographic data were processed using ChemStation software (Hewlett Packard, USA), and dopamine and its metabolites were quantified based on comparison to the peak height of corresponding standards measured on the day of analysis. At the end of the experiments, frozen slices from the brains were examined histologically for appropriate probe placement. Six animals per group were taken in microdialysis study.

### Calculations and Statistics

Two-way analysis of variance (ANOVA) was used to determine the overall significance of the behavioral assay results (locomotor activity). Differences between the control and experimental groups were assessed using Duncan’s test for post hoc analysis. The microdialysis data (for the acute treatment of salsolinol) were measured via one-way ANOVA with repeated measures. The results of chronic administration of salsolinol were analyzed via two-way ANOVA with repeated measures followed (if significant) by Duncan’s test for post hoc analysis.

The results of the biochemical experiments were analyzed via two-way ANOVA followed by Duncan’s test when appropriate. The total catabolic rate of DA was assessed as the ratio of the concentration of the final DA metabolite HVA to that of DA and was expressed as the catabolic rate index ([HVA]/[DA]) × 100 as previously described in detail (Antkiewicz-Michaluk et al. 2001). This index was calculated using the concentrations in each tissue sample (*n* = 7).

## Results

### Behavioral Study

#### The Influence of Acute Salsolinol Administration on l-DOPA-Induced Hyperactivity in Rats

**Fig. 1:**
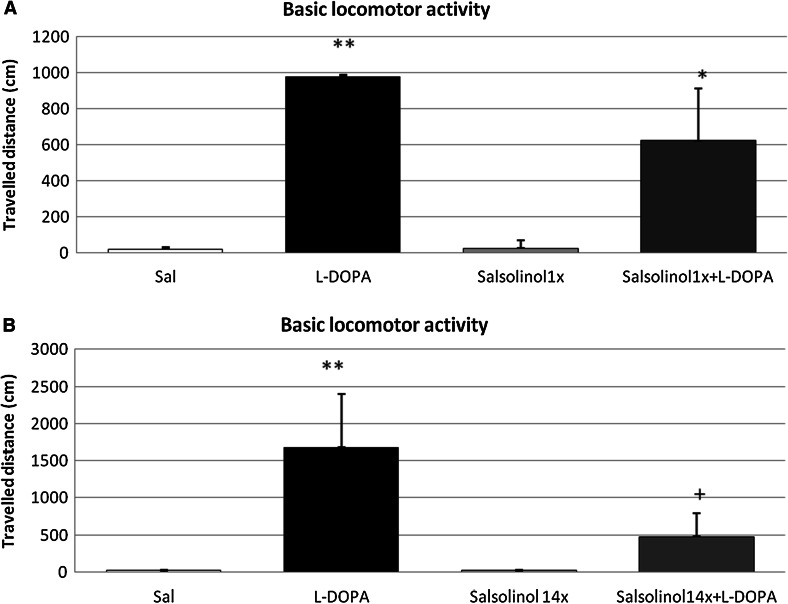
The influence of acute (**a**) and chronic (**b**) administration of salsolinol on l-DOPA-induced changes in the locomotor activity of rats. The rats were placed in actometers and, after 40 min of adaptation, received the specified drugs. Salsolinol was administered at a dose of 100 mg/kg i.p acutely (**a**) or chronically for 14 consecutive days (**b**). In the combined treatment group, l-DOPA (100 mg/kg i.p.) was administered once 15 min after final salsolinol administration. The control rats received a single injection of saline. Next, the measurement was recorded for 30 min. The data are expressed as the means  ±  SEM (*n* = 6). The data were analyzed via two-way ANOVA followed by Duncan’s post hoc test when appropriate. Statistical significance: *P < 0.05, **P < 0.01 versus the control group; ^+^ *P* < 0.05 versus the l-DOPA-treated group

Duncan’s post hoc test showed that acute administration of l-DOPA (100 mg/kg i.p.) induced a significant elevation of horizontal locomotor activity by the rats (*P* < 0.01). However, salsolinol administered alone at a dose of 100 mg/kg i.p. did not change their locomotor activity (Fig. 1a). Similarly, in the combined treatment group, salsolinol did not influence on l-DOPA-induced hyperactivity (Fig. 1a).

#### The Influence of Chronic Salsolinol Administration on l-DOPA-Induced Hyperactivity in Rats

The statistical analysis demonstrated that chronic (14 days) administration of salsolinol (100 mg/kg i.p.) did not change the locomotor activity of rats (Fig. 1b). On the other hand, acute injection of l-DOPA (100 mg/kg i.p.) produced a significant elevation in horizontal locomotor activity of rats (*P* < 0.01). In the combined treatment group, multiple treatment with salsolinol significantly (*P* < 0.05) reduced l-DOPA-induced hyperactivity (Fig. 1b).

### Biochemical Studies

#### Ex Vivo Experiments

##### The Effect of Acute Salsolinol Administration on the l-DOPA-Induced Changes in Dopamine Metabolism in Rat Brain Structures

*Substantia nigra* In the substantia nigra, two-way ANOVA revealed a significant effect of treatment with l-DOPA (*F*[1,27] = 21.85, *P* < 0.01) on the dopamine concentration (Table 1). However, neither the effect of salsolinol (*F*[1,27] = 0.001, N.S.) nor the interaction between salsolinol and l-DOPA was significant (*F*[1,27] = 0.04, N.S.). Duncan’s post hoc analysis demonstrated that acute injection of l-DOPA strongly increased the concentration of dopamine (by approximately 500 %; *P* < 0.01). In contrast, single injection of salsolinol did not alter the dopamine concentration. Additionally, salsolinol administered in combination with l-DOPA did not influence the l-DOPA-induced elevation of the dopamine concentration (Table 1).

**Table 1:** The effects of acute administration of salsolinol on l-DOPA-induced changes on dopamine metabolism in rat brain structures

Treatment		DA (ng/g tissue)	DOPAC (ng/g tissue)	HVA (ng/g tissue)	[HVA]/[DA] × 100
Acute	Acute				
Substantia nigra					
Saline	Saline	889 ± 51	212 ± 34	106 ± 15	12 ± 2
Saline	l-DOPA 100	3985 ± 687**	12,888 ± 2632**	6208 ± 1000**	187 ± 39**
Salsolinol 100	Saline	773 ± 41	185 ± 14	102 ± 7	13 ± 0.8
Salsolinol 100	l-DOPA 100	4146 ± 724**	13,981 ± 1066**	5841 ± 403**	188 ± 57**
*Effect of salsolinol*		*F*_*(1/27)*_ = *0.001* *N.S.*	*F*_*(1/27)*_ = *0.05* *N.S.*	*F*_*(1/27)*_ = *0.04* *N.S.*	*F*_*(1/27)*_ = *0.001* *N.S.*
*Effect of* l-*DOPA*		*F*_*(1/27)*_ = *21.85* *P* < *0.01*	*F*_*(1/27)*_ = *29.4* *P* < *0.01*	*F*_*(1/27)*_ = *40.8* *P* < *0.01*	*F*_*(1/27)*_ = *16.9* *P* < *0.01*
*Interaction of salsolinol* + l-*DOPA*		*F*_*(1/27)*_ = *0.04* *N.S.*	*F*_*(1/27)*_ = *0.05* *N.S.*	*F*_*(1/27)*_ = *0.04* *N.S.*	*F*_*(1/27)*_ = *0.001* *N.S.*
Striatum					
Saline	Saline	9043 ± 430	1238 ± 25	675 ± 46	7.4 ± 0.3
Saline	l-DOPA 100	17,802 ± 1967*	19,266 ± 3690**	10,057 ± 1587**	57 ± 7.9**
Salsolinol 100	Saline	9457 ± 411	1573 ± 109	1017 ± 83	11 ± 0.5
Salsolinol 100	l-DOPA 100	19,351 ± 3151**	22,534 ± 2433**	10,323 ± 503**	62 ± 12**
*Effect of salsolinol*		*F*_*(1/27)*_ = *0.2* *N.S.*	*F*_*(1/27)*_ = *0.3* *N.S.*	*F*_*(1/27)*_ = *0.4* *N.S.*	*F*_*(1/27)*_ = *0.2* *N.S.*
*Effect of* l-*DOPA*		*F*_*(1/27)*_ = *17.8* *P* < *0.01*	*F*_*(1/27)*_ = *30.9* *P* < *0.01*	*F*_*(1/27)*_ = *40.8* *P* < *0.01*	*F*_*(1/27)*_ = *29* *P* < *0.01*
*Interaction of Salsolinol* + l-*DOPA*		*F*_*(1/27)*_ = *0.07* *N.S.*	*F*_*(1/27)*_ = *0.2* *N.S.*	*F*_*(1/27)*_ = *0.001* *N.S.*	*F*_*(1/27)*_ = *0.01* *N.S.*

Salsolinol (100 mg/kg i.p.) or l-DOPA (100 mg/kg i.p.) was acutely administered (100 mg/kg i.p.). In the combined treatment group, l-DOPA (100 mg/kg i.p.) was administered once 15 min after salsolinol administration. The rats were decapitated 2 h after injection. The concentration of dopamine and its metabolites were measured using HPLC. The results are expressed as the means  ±  SEM (*n* = 7–10 animals per group). The data were analyzed via two-way ANOVA followed by Duncan’s test. Statistical significance: * *P* < 0.05, ** *P* < 0.01 versus the control group; ^+^ * P* < 0.05, ^++^ *P* < 0.01 versus the l-DOPA group

Two-way ANOVA demonstrated a significant effect of l-DOPA (*F*[1,27] = 29.4, *P* < 0.01) on the concentration of DOPAC in the substantia nigra (Table 1). In contrast to this, the effect of salsolinol (*F*[1,27] = 0.05, N.S.) and interaction of salsolinol versus l-DOPA (*F*[1,27] = 0.05, N.S.) was not significant. The post hoc analysis indicated that acute administration of l-DOPA produced massive elevation of DOPAC concentration (approximately 6000 %; *P* < 0.01). A similar effect was observed when salsolinol was given combined with l-DOPA (Table 1).

The statistical analysis showed significant effect of l-DOPA (*F*[1,27] = 26.9, *P* < 0.01) on the level of 3-MT (Table 1). However, neither treatment with salsolinol (*F*[1,27] = 0.09, N.S.) nor the interaction between salsolinol and l-DOPA (*F*[1,27] = 0.03, N.S.) was significant (Table 1). The Duncan’s post hoc test showed that l-DOPA induced an increase in the concentration of 3-MT (by approximately 300 %, *P* < 0.01) but that salsolinol did not influence this effect of l-DOPA (Table 1).

In the substantia nigra, two-way ANOVA indicated a significant effect of treatment with l-DOPA (*F*[1,27] = 40.8, *P* < 0.01) on the HVA concentration (Table 1). However, neither treatment with salsolinol (*F*[1,27] = 0.04, N.S.) nor the interaction between salsolinol and l-DOPA was significant (*F*[1,27] = 0.04, N.S.). Post hoc analysis revealed that l-DOPA administration strongly increased the level of HVA (approx. 6000 %, *P* < 0.01), and this effect was similar to that in the combined treatment group (Table 1).

Two-way ANOVA demonstrated a significant effect of l-DOPA (*F*[1,27] = 16.9, *P* < 0.01) on the rate of dopamine metabolism measured as [HVA]/[DA] (Table 1). Statistical analysis revealed neither an effect of salsolinol (*F*[1,27] = 0.001, N.S.) nor an interaction between salsolinol and l-DOPA (*F*[1,27] = 0.001, N.S.) on [HVA]/[DA]. Duncan’s post hoc analysis demonstrated that acute administration of l-DOPA strongly increased the rate of dopamine metabolism (by approximately 1500 %; *P* < 0.01); a similar effect was observed when salsolinol (100 mg/kg) was administered in combination with l-DOPA (Table 1).

#### Striatum

In the striatum, two-way ANOVA indicated a significant effect of treatment with l-DOPA (*F*[1,27] = 17.8, *P* < 0.01) on the dopamine concentration (Table 1). However, neither the effect of salsolinol (*F*[1,27] = 0.2, N.S.) nor the interaction between salsolinol and l-DOPA (*F*[1,27] = 0.07, N.S.) was significant. Duncan’s post hoc test showed that acute administration of l-DOPA increased the dopamine concentration (by approximately 200 %, *P* < 0.01), and acute injection of salsolinol did not alter this effect of l-DOPA (Table 1).

Statistical analysis revealed a significant effect of l-DOPA treatment (*F*[1,27] = 30.9, *P* < 0.01) on the level of DOPAC (Table 1). In contrast, neither the effect of treatment with salsolinol (*F*[1,27] = 0.3, N.S.) nor the interaction between salsolinol and l-DOPA (*F*[1,27] = 0.2, N.S.) was significant. Post hoc analysis demonstrated that treatment with l-DOPA induced a massive elevation in the level of DOPAC (by approximately 1800 %). Acute administration of salsolinol did not alter this effect of l-DOPA (Table 1).

Two-way ANOVA demonstrated a significant effect of acute administration of l-DOPA (*F*[1,27] = 53.3, *P* < 0.01) on the 3-MT concentration in the rat striatum (Table 1). In contrast, neither the effect of salsolinol (*F*[1,27] = 0.3, N.S.) nor the interaction between salsolinol and l-DOPA was significant (*F*[1,27] = 1.6, N.S.). Duncan’s post hoc test showed that acute administration of l-DOPA reduced the level of 3-MT (by approximately 45 %). A similar effect was observed in the combined treatment group.

Statistical analysis indicated a significant effect of l-DOPA treatment (*F*[1,27] = 40.8, *P* < 0.01) on the level of HVA in the rat striatum. In contrast, neither the effect of salsolinol (*F*[1,27] = 0.4, N.S.) nor the interaction between salsolinol and l-DOPA (*F*[1,27] = 0.001, N.S.) was significant. Post hoc analysis demonstrated that treatment with l-DOPA induced a strong increase in the level of HVA (by approximately 1400 %). Salsolinol did not alter this effect of l-DOPA (Table 1).

In the striatum, two-way ANOVA demonstrated a significant effect of l-DOPA (*F*[1,27] = 29, *P* < 0.01) on the rate of dopamine metabolism measured as [HVA]/[DA] (Table 1). Statistical analysis revealed no effect of salsolinol (*F*[1,27] = 0.2, N.S.) nor an interaction between salsolinol and l-DOPA (*F*[1,27] = 0.01, N.S.) on [HVA]/[DA]. Duncan’s post hoc analysis demonstrated that acute administration of l-DOPA increased the rate of dopamine metabolism (by approximately 800 %; *P* < 0.01); similar effect was observed when salsolinol (100 mg/kg) was administered in combination with l-DOPA (by approximately 900 %) (Table 1).

#### The Effect of Chronic Administration of Salsolinol on the l-DOPA-Induced Changes in Dopamine Metabolism in Rat Brain Structures

*Substantia nigra* In the substantia nigra, two-way ANOVA revealed no effect of chronic treatment with salsolinol (*F*[1,24] = 2.4, N.S.) on the dopamine concentration (Table 2). However, statistical analysis indicated a significant effect of treatment with l-DOPA (*F*[1,24] = 30.1, *P* < 0.01) on the level of dopamine. The interaction between chronic administration of salsolinol and acute administration of l-DOPA was not significant (*F*[1,24] = 2.8, N.S.). Duncan’s post hoc test showed that acute administration of l-DOPA induced an increase in the dopamine concentration (by approximately 600 %; *P* < 0.01). Additionally, in the combined treatment group, chronic administration of salsolinol (100 mg/kg) partially blocked the l-DOPA-induced increase in the concentration of dopamine (approximately 300 % increase; *P* < 0.05) (Table 2).

**Table 2:** The effects of chronic administration of salsolinol on l-DOPA-induced changes on dopamine metabolism in rat brain structures

Treatment		DA (ng/g tissue)	DOPAC (ng/g tissue)	HVA (ng/g tissue)	[HVA]/[DA] × 100
Chronic	Acute				
Substantia nigra					
Saline	Saline	844 ± 61	215 ± 18	152 ± 11	18 ± 2
Saline	l-DOPA 100	4775 ± 898**	19,039 ± 3135**	9133 ± 622**	249 ± 60**
Salsolinol 100	Saline	913 ± 59	198 ± 14	146 ± 14	16 ± 1
Salsolinol 100	l-DOPA 100	2996 ± 623*^+^	5372 ± 4089**^+^	9256 ± 1008**	337 ± 87**
*Effect of salsolinol*		*F*_*(1/24)*_ = *2.4* *N.S.*	*F*_*(1/24)*_ = *0.5* *N.S.*	*F*_*(1/24)*_ = *0.01* *N.S.*	*F*_*(1/24)*_ = *0.6* *N.S.*
*Effect of* l-*DOPA*		*F*_*(1/24)*_ = *30.1* *P* < *0.01*	*F*_*(1/24)*_ = *43.5* *P* < *0.01*	*F*_*(1/24)*_ = *233* *P* < *0.01*	*F*_*(1/24)*_ = *26.9* *P* < *0.01*
*Interaction of salsolinol* + l-*DOPA*		*F*_*(1/24)*_ = *2.8* *N.S.*	*F*_*(1/24)*_ = *0.5* *N.S.*	*F*_*(1/24)*_ = *0.01* *N.S.*	*F*_*(1/24)*_ = *0.7* *N.S.*
Striatum					
Saline	Saline	10,439 ± 308	1467 ± 49	1149 ± 31	11 ± 0.5
Saline	l-DOPA 100	21,031 ± 2530**	28,780 ± 3700**	14,858 ± 719**	77 ± 10**
Salsolinol 100	Saline	11,415 ± 394	1789 ± 124	1377 ± 112	12 ± 0.8
Salsolinol 100	l-DOPA 100	16,145 ± 1655*^+^	24,479 ± 5854**	16,492 ± 1180**	106 ± 10**^++^
*Effect of salsolinol*		*F*_*(1/24)*_ = *1.6* *N.S.*	*F*_*(1/24)*_ = *0.3* *N.S.*	*F*_*(1/24)*_ = *1.8* *N.S.*	*F*_*(1/24)*_ = *4.5* *P* < *0.05*
*Effect of* l-*DOPA*		*F*_*(1/24)*_ = *25* *P* < *0.01*	*F*_*(1/24)*_ = *52.1* *P* < *0.01*	*F*_*(1/24)*_ = *432* *P* < *0.01*	*F*_*(1/24)*_ = *128* *P* < *0.01*
*Interaction of salsolinol* + l-*DOPA*		*F*_*(1/24)*_ = *3.6* *N.S.*	*F*_*(1/24)*_ = *0.4* *N.S.*	*F*_*(1/24)*_ = *1.0* *N.S.*	*F*_*(1/24)*_ = *3.9* *N.S*

Salsolinol was administered (100 mg/kg i.p.) chronically for 14 consecutive days. In the combined treatment group, l-DOPA (100 mg/kg i.p.) was administered once 15 min after the final salsolinol administration. The rats were decapitated 2 h after the final injection. The concentration of dopamine and its metabolites were measured using HPLC. The results are expressed as the means  ±  SEM of seven samples (*n* = 7 animals per group). The data were analyzed via two-way ANOVA followed by Duncan’s test. Statistical significance: * *P* < 0.05, ** *P* < 0.01 versus the control group; ^+^ *P* < 0.05, ^++^ *P* < 0.01 versus the l-DOPA group

Two-way ANOVA demonstrated a significant effect of treatment with l-DOPA (*F*[1,24] = 43.5, *P* < 0.01) on the DOPAC concentration in the rat substantia nigra (Table 2). However, neither the effect of chronic administration of salsolinol (*F*[1,24] = 0.5, N.S.) nor the interaction between salsolinol and l-DOPA was significant (F[1,24] = 0.5, N.S.). Post hoc analysis revealed that l-DOPA induced a massive increase in the DOPAC concentration (by approximately 10,000 %; *P* < 0.01) and this effect was partially inhibited by chronic treatment with salsolinol (Table 2).

In the substantia nigra, statistical analysis revealed no effect of chronic treatment with salsolinol (*F*[1,24] = 2.1, N.S.) nor an interaction between chronic administration of salsolinol and acute administration of l-DOPA (*F*[1,24] = 0.4, N.S.) on the level of 3-MT (Table 2). However, the effect of treatment with l-DOPA (*F*[1,24] = 42, *P* < 0.01) on the 3-MT concentration was significant. Duncan’s post hoc test showed that acute administration of l-DOPA induced an increase of 3-MT concentration (by approximately 200 %; *P* < 0.01). A similar effect was observed in the combined treatment group when salsolinol (100 mg/kg) was chronically administered followed by acute injection of l-DOPA (by approximately 200 %; *P* < 0.01) (Table 2).

Two-way ANOVA demonstrated a significant effect of l-DOPA (*F*[1,24] = 233, *P* < 0.01) on the HVA concentration in the rat substantia nigra (Table 2). However, neither the effect of chronic injection of salsolinol (*F*[1,24] = 0.4, N.S.) nor the interaction between salsolinol and l-DOPA was significant (*F*[1,24] = 0.4, N.S.). Post hoc analysis showed that l-DOPA induced a massive increase in the HVA concentration (by approximately 6000 %; *P* < 0.01); chronic treatment with salsolinol did not alter this effect of l-DOPA (Table 2).

In the substantia nigra, two-way ANOVA demonstrated a significant effect of l-DOPA (*F*[1,24] = 26.9, *P* < 0.01) on the rate of dopamine metabolism measured as [HVA]/[DA] (Table 2). Statistical analysis revealed no effect of chronic administration of salsolinol (*F*[1,24] = 0.6, N.S.) nor an interaction between salsolinol and l-DOPA (*F*[1,24] = 0.7, N.S.) on [HVA]/[DA]. Duncan’s post hoc analysis demonstrated that acute administration of l-DOPA strongly increased the rate of dopamine metabolism (by approximately 2200 %; *P* < 0.01), and in the combined group, salsolinol enhanced this effect of l-DOPA (Table 2).

#### Striatum

In the striatum, two-way ANOVA revealed no effect of chronic treatment with salsolinol (*F*[1,24] = 1.6, N.S.) nor any interaction between chronic administration of salsolinol and l-DOPA (*F*[1,24] = 3.6, N.S.) on the dopamine concentration (Table 2). In contrast, the effect of l-DOPA treatment on the level of dopamine was significant (*F*[1,24] = 25, *P* < 0.01). Post hoc analysis indicated that acute administration of l-DOPA increased the level of dopamine (by approximately 200 %, *P* < 0.01); this effect was partially antagonized by chronic treatment with salsolinol (Table 2).

Two-way ANOVA demonstrated a significant effect of l-DOPA (*F*[1,24] = 52.1, *P* < 0.01) on the DOPAC concentration in the rat striatum (Table 2). In contrast, neither the effect of chronic administration of salsolinol (*F*[1,24] = 0.3, N.S.) nor the interaction between salsolinol and l-DOPA was significant (*F*[1,24] = 0.4, N.S.). Post hoc analysis revealed that l-DOPA induced a massive increase in the DOPAC concentration (by approximately 2000 %; *P* < 0.01); chronic treatment with salsolinol did not alter this effect of l-DOPA (Table 2).

Statistical analysis indicated a significant effect of l-DOPA (*F*[1,24] = 163, *P* < 0.01) on the 3-MT concentration in the rat striatum (Table 2). In contrast, neither the effect of chronic injection of salsolinol (*F*[1,24] = 0.6, N.S.) nor the interaction between salsolinol and l-DOPA on the level of 3-MT was significant (*F*[1,24] = 1.4, N.S.). Duncan’s post hoc test showed that l-DOPA decreased the level of 3-MT (by approximately 50 %; *P* < 0.01); this effect was not altered by chronic treatment with salsolinol (Table 2).

Two-way ANOVA showed a significant effect of treatment with l-DOPA (*F*[1,24] = 432, *P* < 0.01) on the HVA concentration (Table 1). However, neither the effect of salsolinol (*F*[1,24] = 1.8, N.S.) nor the interaction between chronic administration of salsolinol and l-DOPA was significant (*F*[1,24] = 1.0, N.S.). Post hoc analysis demonstrated that l-DOPA strongly increased the HVA concentration (by approximately 1100 %; *P* < 0.01); this effect was enhanced by chronic treatment with salsolinol (Table 2).

In the striatum, two-way ANOVA demonstrated a significant effect of chronic administration of salsolinol (*F*[1,24] = 4.5, *P* < 0.05) and acute administration of l-DOPA (*F*[1,24] = 128, *P* < 0.01) on the rate of dopamine metabolism measured as [HVA]/[DA] (Table 2). In contrast, the interaction between salsolinol and l-DOPA was not significant (*F*[1,24] = 3.9, N.S.). Duncan’s post hoc analysis demonstrated that l-DOPA induced an increase in the rate of dopamine metabolism (by approximately 700 %); chronic administration of salsolinol significantly enhanced this effect of l-DOPA (Table 2).

#### The Impact of Acute and Chronic Administration of Salsolinol on l-DOPA Metabolism in the Rat Striatum

One-way ANOVA revealed a significant effect of treatment (*F*[5,40] = 15.22, *P* < 0.01) on the 3-MDOPA concentration in the rat striatum (Table 3). Duncan’s post hoc test indicated that treatment with l-DOPA induced a massive increase in the level of 3-MDOPA (by approximately 30,000-fold; *P* < 0.01); this effect was not altered by either acute or chronic treatment with salsolinol (100 mg/kg i.p.) (Table 3).

**Table 3:** The impact of acute and chronic administration of salsolinol on the l-DOPA-induced increase in the concentration of 3-MDOPA in the rat striatum

Treatment	3-MDOPA (ng/g tissue)
Saline	1.31 ± 0.11
Salsolinol 100 acute	1.46 ± 0.1
Salsolinol 100 chronic	1.39 ± 0.11
l-DOPA 100	17,429 ± 2874**
Salsolinol 100 acute + l-DOPA 100	16,046 ± 1770**
Salsolinol 100 chronic + l-DOPA 100	15,102 ± 3278**
Effect of treatment	*F*_(5/40)_ = 15.22 *P* < 0.01

Salsolinol was administered (100 mg/kg i.p.) acutely or chronically for 14 consecutive days. In the combined treatment group, l-DOPA (100 mg/kg i.p.) was administered once 15 min after the final salsolinol administration. The rats were decapitated 2 h after the final injection. The concentration of 3MDOPA was measured using HPLC. The results are expressed as the means  ±  SEM of six to ten samples. The data were analyzed via one-way ANOVA followed by Duncan’s test. Statistical significance: * *P* < 0.05, ** *P* < 0.01 versus the control group; ^+^ *P* < 0.05, ^++^ *P* < 0.01 versus the l-DOPA group

### In Vivo Microdialysis Study

#### The Effects of Single Administration of Salsolinol on the l-DOPA-Induced Changes in Dopamine Release in the Rat Striatum

**Fig. 2:**
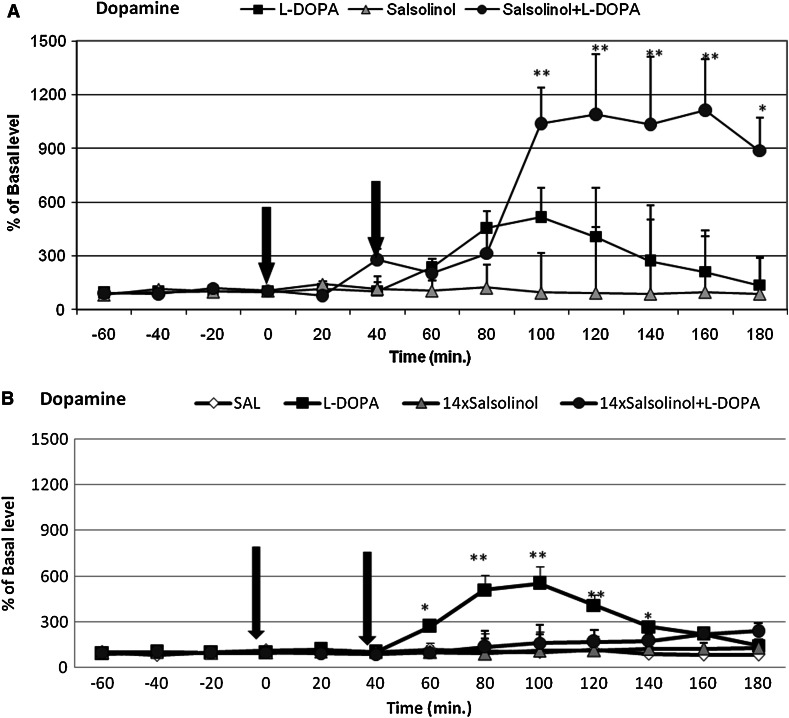
The influence of single (**a**) and chronic (**b**) administration of salsolinol on the l-DOPA-induced changes in dopamine release. Control samples were collected from “−60” to “0”; then, salsolinol (100 mg/kg; at time point “0”) or l-DOPA (100 mg/kg; at time point “40”) was administered i.p. In the combined treatment group, salsolinol was injected 40 min before l-DOPA administration (**a**). As shown in **b**, salsolinol was administered chronically at dose of 100 mg/kg i.p. for 14 consecutive days. In the combined treatment group, l-DOPA (100 mg/kg i.p.) was administered once 40 min after the final salsolinol administration. The control group was treated with saline. Dialysates were collected every 20 min. The concentration of dopamine was measured. The basal level of dopamine in the striatum was 10.6 ± 3.1 pg/20 μl. The data are expressed as the means  ±  SEM (*n* = 6). Statistical significance: *P < 0.05, **P < 0.01 versus the basal level (Duncan’s test)

One-way repeated measures ANOVA revealed a non-significant effect of acute administration of salsolinol (100 mg/kg i.p.) or l-DOPA (100 mg/kg i.p.) on dopamine release into the extracellular space (*F*[2,17] = 2.74, N.S.). However, the effect of time (*F*[12,204] = 5.01, *P* < 0.01) and the interaction between time and treatment was significant (*F*[24,204] =  3.09, *P* < 0.01). Duncan’s post hoc analysis showed that acute l-DOPA administration induced a significant (*P* < 0.05) and persistent increase in dopamine release in the rat striatum (by approximately 500 %) (Fig. 2a). However, salsolinol administered alone did not alter the concentration of dopamine. Post hoc analysis revealed that acute administration of salsolinol combined with l-DOPA potentiated this effect of l-DOPA, inducing the persistent release of dopamine (increased by up to 1200 %) (*P* < 0.01) in the rat striatum (Fig. 2a).

#### The Effects of Chronic Administration of Salsolinol on the l-DOPA-Induced Changes in Dopamine Release in the Rat Striatum

Two-way repeated measures ANOVA revealed a significant effect of treatment 2 (l-DOPA) on dopamine release (*F*[1,17] = 6.73, *P* < 0.05), but neither the effect of chronic administration of salsolinol (treatment 1) (*F*[1,17] = 2.05, N.S.) nor the interaction between salsolinol and l-DOPA (*F*[1,17] = 2.69, N.S.) was significant (Fig. 2b). Statistical analysis demonstrated a significant effect of time (*F*[12,204] = 4.05, *P* < 0.01). Additionally, the interaction between time and treatment 1 (*F*[12,204] = 2.9, *P* < 0.01), between time and treatment 2 (*F*[12,204] = 3.67, *P* < 0.01), and between time, treatment 1, and treatment 2 (*F*[12,204] = 2.4, *P* < 0.01) were significant. Duncan’s test indicated that acute l-DOPA administration induced a significant (*P* < 0.01) and persistent increase in dopamine release in the rat striatum (by up to 500 %); this effect was completely inhibited by chronic administration of salsolinol (Fig. 2b).

## Discussion

The pathogenesis of Parkinson’s disease (PD) is considered to involve both environmental factors and endogenously produced toxins, such as 1BnTIQ and, to a lesser extent, salsolinol. The concept that salsolinol contributes to the pathogenesis of PD arose from the observation that its chemical structure was similar to that of MPTP, a selective dopaminergic neurotoxin which evokes a syndrome resembling the clinical profile of the disease in humans and animals (Langston et al. 1983). In fact, our previous clinical studies revealed that the concentration of salsolinol in the CSF of patients with advanced parkinsonism was significantly increased and that this increase correlated to the state of dementia rather than to the progression of parkinsonism (Antkiewicz-Michaluk et al. 1997). The latter finding was in line with studies showing that exogenous salsolinol crossed the blood–brain barrier and interacted with dopamine receptors in the brain (Antkiewicz-Michaluk et al. 2000a). Further, it was demonstrated that salsolinol displaced [^3^H]apomorphine from its binding sites on dopamine D1 and D2 receptors at an efficacy similar to that of dopamine, and in behavioral studies, salsolinol inhibited apomorphine-stimulated locomotor activity (Antkiewicz-Michaluk et al. 2000a). The ability of salsolinol to interfere with the agonist binding sites on dopamine receptors, thereby inhibiting their function, suggests that this compound can attenuate dopaminergic neurotransmission at sites other than those to which classical neuroleptics bind. In fact, the activity of salsolinol as an endogenous neuroleptic is quite different from that of typical neuroleptics, and even at a high dose, salsolinol did not induce catalepsy (Antkiewicz-Michaluk et al. 2000a).

The primary finding of the present study is that chronic administration of the endogenous compound salsolinol to rats reversed the enhancement of l-DOPA-induced behavioral hyperactivity, dopamine metabolism, and dopamine release in dopaminergic brain structures. The neuroleptic-like activity of salsolinol may be responsible for certain motor deficits observed in chronically salsolinol-treated animals and for the attenuation of l-DOPA-induced locomotor hyperactivity (Fig. 1). Biochemical ex vivo studies showed that administration of l-DOPA (100 mg/kg i.p.), a precursor of dopamine, causes a significant increase in the dopamine concentration, dopamine metabolism, and the concentration of all dopamine metabolites in extrapyramidal brain structures, including the substantia nigra and the striatum. Comparing the results from the behavioral and biochemical experiments (both ex vivo and in vivo), we concluded that salsolinol does not always act in the same direction. A single dose of salsolinol (100 mg/kg) did not change l-DOPA-mediated hyperactivity based on the behavioral assay (Fig. 1a) as well as did not exert any effect on the l-DOPA-mediated increase in dopamine metabolism in the examined brain structures (Table 1), despite significantly potentiating l-DOPA-mediated dopamine release in the rat striatum (Fig. 2a). These data are in agreement with the previously described profile of salsolinol activity in the brain. As noted above, salsolinol displays moderate affinity to the D1 and D2 dopamine receptors and acts as an antagonist. The results of the in vivo microdialysis experiments analyzing the influence of salsolinol on l-DOPA-mediated dopamine release are of particular importance. Notably, auto- and pre-synaptic D2 dopamine receptors (inhibitory receptors) are directly responsible for the regulation of the rate of dopamine metabolism and release. Somatodendritic D2 autoreceptors in the SN inhibit the excitability of dopamine neurons and modulate the firing rate by activating a hyperpolarizing potassium current (Aghajanian and Bunney 1977) and presynaptic autoreceptors on nerve terminals (in the striatum) regulate dopaminergic transmission by inhibiting dopamine release, synthesis, and uptake (Cass and Gerhardt 1994; Truong et al. 2004; Seeman and Van Tol 1994). In fact, by acting as an antagonist of dopamine receptors, salsolinol may induce, potentiation of l-DOPA-induced dopamine release. Additionally, as shown by Homicsko et al. (2003), salsolinol can be displaced from its binding sites by dopamine, l-DOPA, apomorphine, benserazide, and carbidopa. Many of these displacers target aromatic amino acid decarboxylase (AADC), although salsolinol does not directly affect AADC activity (Toth et al. 2002). These authors concluded that there are three major candidates for salsolinol binding sites: AADC, vesicular monoamine transporter, and an unidentified receptor that also displays high affinity to dopamine (Homicsko et al. 2003; Toth et al. 2002).

Interestingly, different effects were observed after chronic salsolinol (100 mg/kg) treatment. In the behavioral assay (Fig. 1b) and ex vivo biochemical analysis, chronic salsolinol administration was demonstrated to significantly reduce the l-DOPA-induced increase in the dopamine concentration in the substantia nigra and the striatum (Table 2). Similarly, the DOPAC concentration was reduced in the animals co-treated with salsolinol and l-DOPA in comparison to those treated with l-DOPA alone; however, the level of HVA was not different (Table 2). In that case, the [HVA]/[DA] index, which was used as an indicator of the rate of dopamine metabolism, was significantly increased in the striatum. These results suggest that chronic administration of salsolinol impaired both dopamine synthesis and storage in neurons. The present results are in agreement with those of our previous experiments, in which we showed that chronic administration of salsolinol caused moderate damage to striatal dopaminergic neurons in rats (Antkiewicz-Michaluk et al. 2000b; Lorenc-Koci et al. 2000). Moreover, other authors have demonstrated that salsolinol may act as an inhibitor of both tyrosine hydroxylase, a rate-limiting enzyme in dopamine synthesis, (Almas et al. 1992; Minami et al. 1992) and MAO activity (Maruyama et al. 1993). Salsolinol has also been considered to inhibit catecholamine storage in the rat brain (Heikkila et al. 1971) and to impair the function of vesicular monoamine transporter-2 (VMAT2) at dopaminergic terminals (Naoi et al. 2004). In fact, our microdialysis study has shown that chronic salsolinol administration, in contrast to its acute administration, completely antagonized l-DOPA-evoked dopamine release, suggesting that salsolinol dysregulated the process of dopamine storage and dopamine release into the extracellular space (Fig. 2b). Interestingly, the in vitro studies of other authors may partially explain the toxic mechanism of action of salsolinol. Storch et al. (2000) concluded that salsolinol was toxic to dopaminergic neuroblastoma SH-SY5Y cells by blocking the cellular energy supply via inhibition of mitochondrial complex II activity. Others found that incubation of SH-SY5Y cells in salsolinol resulted in a rapid dose- and time-dependent decrease in the intracellular level of ATP and in maximal turnover of glycolysis without compensating for this rapid energy depletion (Morikawa et al. 1998). As recently demonstrated by Brown et al. (2013), salsolinol exerts apoptotic effects via caspase-3 upregulation and induces a reduction in the levels of BDNF and its signaling protein p-CREB. Wanpen et al. (2004) demonstrated that salsolinol produced both apoptosis (by causing caspase-3 activation) and oxidative stress (by depleting reduced GSH levels and by increasing ROS production) in dopaminergic SH-SY5Y cells. The same research group demonstrated that salsolinol induced DNA damage, nuclear DNA fragmentation, decrease in TRPC1 protein levels and Ca^2+^ influx, and this way decrease in cell viability (Shavali et al. 2003; Bollimuntha et al. 2006).

Based on these in vitro studies it is clear that salsolinol may act toxic on the dopaminergic neurons by complex mechanisms. It is known that in vitro experiments are not always in agreement with in vivo research. Generally, it belongs to take under consideration that in vitro toxicity can differ from in vivo obtained results, which are dependent on complicated interactions among different neurotransmitters in the brain. Additionally, we investigated the effect of acute and chronic salsolinol administration on COMT activity, which was analyzed indirectly as the concentration of the l-DOPA metabolite 3-MDOPA, after l-DOPA administration (Table 3). l-DOPA is well understood as a direct substrate of COMT, and its metabolism leads to the production of 3-MDOPA. COMT is responsible for both the metabolic inactivation of extraneuronal dopamine via its conversion to 3-MT and the metabolism of l-DOPA into 3-MDOPA (Kopin 1985; Antkiewicz-Michaluk et al. 2001). Finally, the results of the present study indicated that neither acute nor chronic administration of salsolinol affected COMT activity in the brain.

Combining the results of our behavioral, biochemical ex vivo and in vivo studies, we suggest that chronic, but not acute, administration of salsolinol disrupted the function of dopaminergic neurons and significantly impaired the effects of l-DOPA. Therefore, we propose that an elevated salsolinol level in parkinsonian patients may represent a serious risk factor of the clinical efficacy of l-DOPA therapy.

## Abbreviations

AADC: Aromatic amino acid decarboxylase
BDNF: Brain-derived neurotrofic factor
1BnTIQ: 1-Benzyl-1,2,3,4-tetrahydroisoquinoline
COMT: Catechol-*O*-methyltransferase
CREB: Cyclic AMP response element-binding protein
CSF: Cerebrospinal fluid
DAT: Dopamine transporter
DOPAC: 3,4-Dihydroxyphenylacetic acid
HVA: Homovanillic acid
L-DOPA: 3,4-Dihydroxy-*l*-phenylalanine
MAO: Enzyme monoamine oxidase
3-MT: 3-Methoxytyramine
3MDOPA: 3-Methoxy-DOPA
MPTP: 1-Methyl-4-phenyl-1,2,3,6-tetrahydropyridine
PD: Parkinson’s disease
Salsolinol: 1-Methyl-6,7-dihydroxy-1,2,3,4-tetrahydroisoquinoline
SN: Substantia nigra
VMAT2: Vesicular monoamine transporter-2
